# Coming Full Circle: How Health Worker Motivation and Performance in Results-Based Financing Arrangements Hinges on Strong and Adaptive Health Systems

**DOI:** 10.15171/ijhpm.2018.98

**Published:** 2018-10-29

**Authors:** Sumit Kane, Crecentia Gandidzanwa, Ronald Mutasa, Irene Moyo, Chenjerai Sismayi, Patron Mafaune, Marjolein Dieleman

**Affiliations:** ^1^KIT Royal Tropical Institute, Amsterdam, The Netherlands.; ^2^Nossal Institute for Global Health, Melbourne School of Population and Global Health, University of Melbourne, Melbourne, VIC, Australia.; ^3^Independent Consultant.; ^4^World Bank Group, Washington, DC, USA.; ^5^Ministry of Health and Child Care, Government of Zimbabwe, Harare, Zimbabwe.

**Keywords:** Performance Based Financing, Results Based Financing, Motivation, Health Sector Reform, Organisational Change, Zimbabwe

## Abstract

**Background:** This paper presents findings from a study which sought to understand why health workers working under the results-based financing (RBF) arrangements in Zimbabwe reported being satisfied with the improvements in working conditions and compensation, but paradoxically reported lower motivation levels compared to those not working under RBF arrangements.

**Methods:** A qualitative study was conducted amongst health workers and managers working in health facilities that were implementing the RBF arrangements and those that were not. Through purposeful sampling, 4 facilities in RBF implementing districts that reported poor motivation and satisfaction, were included as study sites. Four facilities located in non-RBF districts which reported high motivation and satisfaction were also included. Data was collected through in-depth interviews and analyzed using the framework approach.

**Results:** Results based financing arrangements introduce a wide range of new institutional arrangements, roles, tasks, and ways of doing things, for facility staff, facility managers and, district and provincial health management teams. Findings reveal that insufficient preparedness of people and processes for this change, constrained managers and workers performance. Results based financing arrangements introduce explicit and tacit changes, including but not limited to, incentive logics, in the system. Findings show that unless systematic efforts are made to enable the absorption of these changes in the system: eg, through reconfiguring the decision space available at various levels, through clarification of accountability relationships, through building personnel and process capacities, before instituting changes, the full potential of the RBF arrangements cannot be realised.

**Conclusion:** Our study demonstrates the importance of analysing existing institutional, management and governance arrangements and capabilities and taking these into account when designing and implementing RBF interventions. Introducing RBF arrangements cannot alone overcome chronic systemic weaknesses. For a system wide change, as RBF arguably is, to be effected, explicit organisational change management processes need to be put in place, across the system. Carefully designed processes, which take into account the interest and willingness of various actors to change, and which are cognizant of and constructively engage with potential bottlenecks and points of resistance, should accompany any health system change initiative.

## Background

Several low- and middle-income countries are turning to results-based financing (RBF) arrangements as a means to achieve their universal health coverage related ambitions. Others, some of the early adopters of the RBF arrangements, are in the process of scaling up. The literature reveals a vibrant debate between proponents and doubters of the RBF approach. Interestingly, this debate is not restricted to academia, but has wide participation of policy-makers, practitioners, and researchers. The questions under debate are wide ranging; for example: some scholars are unpacking the origins and political economy leanings of RBF,^1^ some are critically examining the key assumptions underpinning RBF,^2^ and others are examining operational and implementation challenges to inform policy and to improve practice.^3-6^

This study adds to this growing body of empirical examination of the key assumptions underpinning RBF; it does so in light of the operational and implementation ground realities. A recent survey by Nguyen et al,^7^ done as part of an impact evaluation of the RBF reform in Zimbabwe, found lower levels of motivation amongst health workers working under RBF arrangements. We present the results of a qualitative study that sought to explain these findings. The insights gained from this study will be useful beyond the study settings; they can help both, countries that are contemplating the introduction of RBF, and those in the process of scaling up.

Zimbabwe, with support from the World Bank, initiated a reform process which introduced RBF for health arrangements in 2011. While RBF entails some changes across the 6 building blocks of the health system, it primarily works through targeting financing, leadership and governance and some human resource processes in the health system. In contrast to the traditional input-based financing, RBF is an output-based financing arrangement in which a principal entity purchases services from a recipient conditional to quality and after verification of declared results.^8,9^ Within RBF arrangements, health workers individually, and collectively as a facility, are rewarded based on their performance measured against pre-agreed targets. Facilities are given the autonomy to use the facility level rewards to fund improvements in service delivery. The intervention logic of RBF arrangements is that a combination of monetary rewards based on results, together with the autonomy to use these funds, leads to health facilities improving their working conditions, and health workers being motivated to perform and to deliver quality care (see p24).^10^ RBF arrangements are not meant to replace existing systems, but rather to facilitate and/or enforce policies/processes that are identified as being not well functioning. For example, in the context of Zimbabwe, through inclusion as conditions in the contracts, RBF seeks to ensure that each contracted facility has a functional health centre committee (HCC), and that facilities do not charge any fees for services; similarly, it seeks to encourage the conduct of supervision visits.

There have been notable improvements in the coverage and quality of services since the introduction of RBF arrangements in parts of Zimbabwe; these are detailed in an evaluation conducted by the World Bank in 2016.^11^ However, insights from the survey done by Nguyen et al^7^ revealed that while those health workers under the RBF arrangements were satisfied with the improvements in working conditions and compensation, their motivation levels were lower. Nguyen and colleagues’^7^ study measured and compared satisfaction and motivation amongst frontline health workers working in RBF intervention areas, and those not. Survey measurements were conducted at baseline, before the RBF intervention began, and again after 30 months. Motivation was studied as an aggregate of 8 constructs, namely: teamwork, autonomy, recognition, self-concept, change in facility, work environment, leadership of facilities, and well-being. While motivation levels fell across all 8 constructs, the poorest outcomes were for leadership of facilities, teamwork, and well-being This fall in the motivation of frontline health workers, occurring with 30 months of initiation of the RBF intervention, was a matter of concern; it was also contrary to the RBF intervention logic. Nguyen and colleagues^7^ study showed that not only did the motivation among those working under RBF fall, it was significantly lower than in the non-RBF areas. Thus, the question that this study sought to answer was ‘Why did health workers working under RBF arrangements report lower levels of motivation despite being satisfied with the working conditions and receiving better compensation?’ The objective of this study was therefore to gain insight into the reasons behind this fall in motivation, and to use this insight to inform policy and practice.

These findings about health workers experience of RBF arrangements are however to some extent in line with what has been recently reported from Zambia by Shen et al.^12^ More broadly, Asiri et al^13^ in their recent review of research on factors influencing motivation and performance of health workers, argue that managers’ ability to provide support, facilitate team work, manage conflicts and negotiate with their superiors and other stakeholders, shapes the motivation and performance of the staff they manage. Asiri et al^13^ add that managers do not function in a vacuum – that the manager’s ability to act depends, among others, on their leadership and management competencies, decision-making space and tools and support they themselves have access to. In this paper, we draw on this literature to show that for the RBF premise to hold, ie, for frontline health workers to be able to exercise autonomy and work together to achieve results, requires that conditions are created, and health facility managers are supported and enabled, to exercise leadership to motivate their teams to work together. We critically examine the RBF intervention in Zimbabwe to expose the key factors that influenced health facility manager’s ability (or not) to exercise leadership, improve teamwork to improve their facility’s performance.

## Methods

### Study Design

A qualitative exploratory study was conducted between June 2015 and October 2015 to explore factors influencing health facility managers’ ability to exercise leadership and to improve teamwork-the main determinants responsible for the decline in motivation. The performance-based financing framework of the Health Results Innovations Trust Fund (HRITF) was chosen as the Apriori conceptual framework to guide the inquiry.^14^ This framework was chosen because it comprehensively articulates the potential contextual factors that could influence the implementation of RBF interventions. It was also chosen because it is used in Zimbabwe to guide inquiries and analyses geared towards identifying areas for learning about and improving RBF design and implementation. According to the framework, a variety of factors, operating across several contextual levels, influence RBF interventions: intervention context, health system context, health facility context, geographic context, community context, and the political context. Adopting these categories of contextual factors to guide our inquiry allowed us to take a broad and comprehensive view in the questions we asked – such an approach was particularly appropriate for the exploratory nature of the inquiry.

### Sampling

Sampling was purposeful. Four facilities located in RBF-districts that had reported poor motivation and satisfaction during the impact evaluation, with results ranging from 67%-80% of facility staff being demotivated, were included as study sites. We included 4 facilities located in non-RBF districts which reported high motivation and satisfaction so as to identify if similar factors influenced leadership and teamwork in these settings. These were included to enrich our data and to improve sense-making through comparing and contrasting across the 2 settings. Cadres to be interviewed were purposively selected with the aim of including all types of cadres affected by the RBF arrangement in the facility; members of the Provincial Health Executive (PHE) and District Health Executive (DHE) were also included as key informants with a view to gain insight into broader contextual influences. Table shows the respondents who were interviewed; details of the study districts and sites where the respondents hailed from, have been withheld to maintain confidentiality. Further details of the context of the districts where the study was conducted can be found in Nguyen et al^7^ and in the evaluation conducted by the World Bank.^10,14^

**Table:** Study Participants

**Cadre Interviewed**	**Number**	
	**RBF**	**Non-RBF**
PHE	3	
DHE	6	7
RGN	2	3
PCN	6	5
EHT	1	2
Nurse aid	2	4
Primary counsellors	1	2
General hand	2	3
Total	49	

Abbreviations: RBF, results-based financing; PHE, provincial health executive; DHE, district health executive; RGN, registered general nurse; PCN, primary care nurse; EHT, environmental health technician.

### Data Collection and Analysis

Topic guides were developed to conduct in-depth interviews with managers/supervisors at district and provincial levels, and with managers (nurse in charge) and health workers at facility level. These topic guides were refined, and probes were added to include emerging themes as the data collection (and simultaneous preliminary analysis) proceeded. All interviews were carried out by 2 members of the research team (CG, IM); all interviews were conducted in English, were recorded using voice recorders, and audio files were transcribed verbatim. Data were collected over a 2-week period from 19 to 31 October 2015. The processes of analysis of data began in tandem with data collection; at the end of each day of data collection the researchers debriefed and shared field notes, to note the emerging analytical themes. Once data collection was completed, and audio files transcribed, a framework approach^15^ was used to analyse the transcripts. This meant that the research team analysed data through a process of reading, re-reading and discussing, followed by charting and sorting of key issues and themes that emerged from the data. This was an iterative, deliberative process that involved holding the findings and their context against theory, to proffer explanations.

Transcripts were coded by 2 researchers independently in NVivo software (v 11); they were coded to nodes derived from the inquiry framework, and from the themes that emerged during data collection and analysis. Triangulation of findings across facilities, participant categories, and data collection methods allowed a thorough understanding of experiences, and of the factors shaping these, to emerge.

## Results

This section presents key findings about what all influenced the facility manager’s ability to effectively lead their staff and to motivate them to work together to achieve results. Findings in the form of specific themes are organised under the broad lines of the various contexts articulated in the HRITF framework^14^: health system context, health facility and individual context, and the local context: local politics, community characteristics and geography.

However, the headings in this section do not map exactly to the HRITF framework categories. They instead reflect what emerged from the interviews as being important; for instance, the health system and health facility contextual domains elicited the most responses and insights from the respondents. For example, the community domain elicited some insights; however, these overlapped with the political-economy domain, but only with the local politics and not the broader political-economy as articulated in HRITF framework. Similarly, while in the HRITF framework, geography is subsumed in the community context – our findings reveal that geographical considerations shape health workers experience in multiple ways, linking across community, health systems, and health facility contexts. Hence, geography is presented as a separate heading in this section. The structure of the findings section thus reflects what we found and how much of it. This divergence from how things are imagined in the HRITF framework is understandable given that study respondents were facility level frontline staff and frontline managers who are more likely to relate their experiences to the immediate facility-system-community contexts, and not to the broader political-economy context. Findings illustrate that the local and more mundane implementation dynamics are as important as the larger contextual influences, perhaps even more important, to comprehensively understand the implementation processes around RBF.

### Health System Context

The facility manager’s ability to effectively lead their staff, to motivate them to work together to achieve results, irrespective of the broader incentive arrangements (results based or unlinked), was to a large extent explained by existing health system management arrangements which were either yet to be addressed by or were beyond the ambit of influence of the RBF arrangements.

#### Weaknesses in Managing Change: Deployment of Registered General Nurses as Facility In-Charge

The lack of a systematic process for managing change in the management arrangements in RBF facilities, specifically the deployment of newly qualified and relatively inexperienced registered general nurses (RGNs) as facility in-charge, has led to friction amongst, and demotivation of facility level staff. According to policy it is the RGN who is in-charge by virtue of holding higher qualifications. But entrenched informal professional hierarchies in the health system, seriously constrained the ability of in-charges of health facilities, to lead their teams well. For instance, a common issue pertained to RGNs being appointed as nurse in-charge (NIC); this involved them superseding existing cadres. RGNs were often younger and less experienced than the other staff they oversaw in their capacity as NIC. This, perhaps inevitably, created a difficult environment for RGNs in both RBF and non-RBF facilities. This, however, played out stronger in RBF-facilities where they were required to exercise leadership and foster teamwork for the facility targets to be achieved. It further came to the fore when the often younger and less experience RGNs also did not have a good understanding of service delivery and/or RBF arrangements. When RGNs were deployed to primary care facilities, other established facility staff struggled to accept them as the new boss. At some facilities the primary care nurses (PCNs) who were in-charge prior to the arrival of the RGN, and had years of experience, refused to cooperate in orienting the new boss. That many PCNs felt that now they were being considered not good enough to do what they had always done, further complicated matters; as the following quotes illustrate, the system fell woefully short in managing this major change in facility level management arrangements.

*“There was a time whereby we felt we are under estimated by this other cadre of ours (by RGNs and others). I do almost the same duties despite the fact they have higher training. This was mostly in 2013 and 2014 … we almost felt like leaving”* [PCN].

*“In 2012 I was selected to be focal person of TB programme. After I came from upskilling I was told an RGN should be the focal person. Such things make us feel that we are made to work when it is convenient for them but when RGNs come we are moved” *[PCN].

This was also the case for the district medical officers (DMOs) who were also members of the DHE. For instance, most DMOs in the selected districts were young (3 of the 4 were just about 30 years old); they had limited public health and leadership experience. As the following quote illustrates, at some places, DHE members were not trained in RBF and yet they were supposed to advise and guide the health facilities. The poor support from higher management in preparing facility managers to deal with change in general and in particular to support various cadres to cope with and work within the new RBF arrangements was a source of frustration and a demotivator for many health workers.

*“Training for nurses, doctors or EHTs [environmental health technicians] has no management aspects. Maybe that is one thing that needs to be included in the curriculum. For me my seniors are the ones who have been guiding me through it. Everyone learns on the job there is no training organised to help you cope on the job. Even doctors need management training because in the last 3 years it has not happened”* [DMO].

DHEs have the mandate to oversee all health facility teams, including to effect changes in the facility leadership as appropriate. DHEs are also required to actively manage the change process to ensure that transitions do not negatively impact service delivery. However, the above instance signals that these change management process did not go well, at both DHE and facility levels. DMOs across both RBF and non-RBF contexts had limited public health and leadership experience and capacity. However, the challenge of implementing a reform as complex as RBF meant that the DMOs in the RBF districts had greater difficulties in supporting the facilities they oversaw.

#### Lack of Clarity on Roles and Lines of Reporting

Lack of clarity about roles, responsibilities, and lines of reporting, either because of ambiguities in RBF related institutional arrangements, due to poor direction from higher-ups, or sometimes the result of how these were interpreted and applied at the local level, led to tensions in inter-cadre and interpersonal relations. It affected the ability of facility managers to exercise leadership and to foster teamwork. This problem was systemic and played out in both RBF and non-RBF settings. For instance, as the following excerpts from interviews with an RGN, a PCN and a PHE show, there were ambiguities in even the most routine and in some ways mundane matters like the overseeing of duty rosters; these were enough to trigger resentment and to surface broader underlying inter-cadre animosities.

*“They had clashes on roles and responsibilities. The doctor was planning daily duties and allocating duties for nurses instead of the NIC. Two people were causing confusion to the members of staff. Factions were created as a result. The nurse aides, general hands and other nurses were on the side of the doctor and the other staff on the side of the NIC”* [RGN].

Similarly, while provincial and district managers are clear regarding roles of and relationship between the EHT and NIC in health facilities, other institutional arrangements were such that they left room for friction, no matter the broader incentive arrangements. EHTs reported to parallel structures while the head of facility was the NIC. The problem came to the fore when RBF arrangements came into force; meetings had to be held and the challenge was who should chair the meetings. At facility level, in most cases, because there was no clarity on these matters, it led to tensions between nurses and EHTs with regard to who was really in charge. The EHTs tend to be older, often male, and on the job for longer, whereas the RGNs tended to be women, younger and newer. In some facilities the EHT refused to take instructions from the RGN/NIC; this was particularly problematic when the NIC was a PCN and thus less qualified than the EHT. Communication between EHTs and nurses was poor to the extent that an EHT would go for field visits without informing the NIC. In such cases the NIC would mark the EHT as absent from duty resulting in EHTs losing out on RBF incentives, as illustrated by this quote from a PCN:

*“For example, an EHT at a clinic went for study leave without communicating with the NIC at his clinic but the district knew he had gone for study leave. When the RBF funds came he also wanted the incentives, but the staff refused since he was on study leave. He complained to the district about the issue and this was addressed by the DHE. It was resolved that he be paid the number of days he worked at the facility and should also strengthen communication with his colleagues. This was last year 2014. These challenges have been in existence for long but now they are exposed because of RBF which requires team effort”* [PCN].

We found that these ambiguities in lines of reporting and accountability at the facility level, particularly concerning the NIC and the EHT, were problematic. These ambiguities were a constant source of discord and demotivation amongst facility level health workers. Our findings show that these problems are systemic, and they cut across RBF and non-RBF districts; however, we found that this lack of clarity was particularly acute in RBF districts. This appeared to be due to design issues in the RBF institutional arrangements, or to poor direction from higher-ups, or sometimes the result of how these were interpreted and applied at the local level. As the following quote shows, this was further complicated by the fact that often the district and provincial managers approached these problematic institutional and relational arrangements as being merely interpersonal problems, and as something that had to be and could be managed as such.

*“The EHTs work is not centered on the clinics. He has to go out and collect the samples and come back. But the nurses are always at the clinic. They need to work together as a team complaining is not warranted”* [PHE].

#### DHE Capacity to Coach and Mentor, Supports or Hampers Health Facility Performance

Findings revealed that health facility staff at an RBF site found supportive supervision as being helpful; they find being mentored, guided, motivated and assisted in conflict resolution and problem solving, very important. As the following excerpt from an interview with a DMO illustrates, this is an aspect of the system that has improved substantially since the institution of RBF arrangements.

*“DHE is getting funds from RBF for fuel and servicing of cars, now we are able to conduct supervision more regularly and verifying data. Whatever gaps were there before they are becoming more noticeable. There has also been an increase in the programmes that have been going to the clinics eg, decentralisation of antiretroviral therapy … which we cannot attribute to RBF. RBF has given us an opportunity to look at the gaps at the health facility. So even if RBF is finished we should continue the monitoring”* [DMO].

However, local and individual level weaknesses in the implementation of what appear otherwise to be robust institutional arrangements (eg, related to supervision), continue to undermine staff motivation, irrespective of the broader incentive framework. For instance, and not unexpectedly, in both RBF and non-RBF sites, whenever DHE’s supportive supervision was focused on fault finding and lacking confidentiality, as the following quote from a PCN illustrates, it discouraged those who were thus supervised.

*“At times lack of praise by the DHE affects our performance. At times the DHE comments using discouraging words. If you are praised, you become happy”* [PCN].

A DMO candidly accepted that *‘Or maybe it’s because on some occasions we shout at them for poor performance but it’s not so many times.’* She and others added that given the multiple responsibilities they had, and the additional responsibilities they now had under the RBF arrangements, meant that there was much pressure on their time – preventing them to perform their jobs optimally.

#### Temptation for Higher-Ups to Override Local Resource Allocation Decisions

The RBF arrangements explicitly seek to increase autonomy at facility level, and health facility staff (including and together with facility managers in lead) are expected to set their own priorities, including in matters pertaining to allocation of resources. However, facility level managers and staff feel that there is too much interference from the district and the province levels, and that this undermines their ability to achieve their targets. On the other hand, as the following quote clearly shows, some managers at district and provincial level felt that they had the right to intervene – to ensure that lower level facilities were operating within guidelines, and not abusing their autonomy.

*“We once had a provincial visit to the clinic and they concluded that the clinics should not be given absolute autonomy because they can abuse funds e.g. buying expensive items and giving each other allowances”* [DMO].

These competing interests, and the ability of higher-ups (PHE/DHE) to override local resource allocation decisions undermined the ability of facility level managers to exercise their leadership and to foster teamwork, particularly in RBF facilities. In non-RBF facilities with the traditional input-based financing and no rewards to be had for achieving target, this was not an issue as the facility level managers expected higher ups to intervene, and there was no price to be paid for any decisions that were contravened by higher ups.

#### Administrative Constraints Undermine Gains Made Through Structural Changes (Changes Like RBF)

Administrative processes which are beyond the control of the facility manager, eg, delays in the receipt of incentive monies from the fund holder, hinder the facility manager’s ability to credibly lead her team and to foster teamwork. It also delays the execution of jointly agreed plans and translates into loss of potential financial gains for staff – thus, as the quotes below illustrates, frustrating and demotivating the staff.

*“Our plan was to electrify the clinic but the money we received was too late and too little, so we sacrificed even our incentives for electrification so that we attract mothers to come and deliver here”* [GH].

Facility level managers and staff reported this as a major concern; they pointed out that delays in disbursements were a regular problem; and that the processes to collect the incentive monies were tedious and that requests (forms) were returned for trivial mistakes. All this undermined the ability of facility managers to lead and motivate their staff to work as a team to achieve results.

#### System Wide Staffing Shortages

While RBF arrangements have goaded and motivated health workers to improve access to services, the system wide shortfall in capacity (both infrastructure and human resource) has meant that this expansion of access is perhaps occurring at the expense of quality of care. Staff deployment to some facilities has not been consistent with the catchment population. For example, a facility in a non-RBF district had 3 nurses for a population of about 3000, yet another facility in an RBF district had only 2 nurses serving a population of over 6000. Similarly, many facilities experienced a double burden of staff shortages and high work load due to the many programmes that had been decentralised from higher levels of care in an effort to offer comprehensive primary care services. These human and material constraints undermined the quality of care.

Our findings signpost that RBF related changes, if implemented without concomitant appropriate human resource redeployments and reforms by the ministry of health, can undermine the ability of health facility teams to fulfil their responsibilities. For example, increased demand for services can create situations where there is mismatch between the population being served and the workload – leading to, as the quote below highlights, frustration, and low motivation.

*“We do not assess the patients properly ... which is frustrating because we may misdiagnose ...” *[RGN].

### Health Facility and Individual Context

While the above health system level issues affect the ability of facility level managers to effectively lead their staff, and to motivate them to work together to achieve results, the study found some local facility context level factors that could also independently influence the manager’s abilities.

#### Incentives as Rallying Points for Teamwork

In the RBF districts, teamwork was uniquely fostered by the clear and shared benefits of working as a team to achieve targets and to earn higher incentives. However, in the non-RBF sites there was no such rallying point that would foster teamwork; here teamwork was not seen as being important as health facilities received a fixed compensation. One PCN at one facility in a non-RBF district pointed that the ubiquity of delays in the submission of documentation to the district (to submit regular reports all staff would need to work together to complete it), in some ways best symbolised the poor state of teamwork in her context. In contrast, under RBF arrangements, given the collective expectation of having additional financial resources, for their facilities and for themselves alike, staff have become more organized and work together to improve documentation (reporting is one of the results within the RBF program).

#### Individual Manager’s Capacity and Preparedness to Fulfil Management Functions

In both, RBF and non-RBF facilities, health workers reported problems with the facility leadership, and sometimes district level leadership, as being a key demotivator. Although not specifically mentioned, the importance of good leadership capacities and capacities to stimulate teamwork are at the heart of good management practice; and the lack of this is likely to have had a stronger impact on the RBF facilities, given the shift from centralized decision making to local level decision making regarding the allocation and use of funds. Similarly, leadership is key to ensuring collaboration within teams, and to enable smooth interactions with other stakeholders. Leadership related problems that the staff referred to were often related to individual managers, and their capacities. For instance, many facility staff highlighted issues of favouritism in the selection of staff to attend trainings; this was an important source of discontent and demotivation amongst many health workers. Besides the learning, and the time off from work, staff viewed the trainings as an opportunity to earn some extra income, given the allowances that went with the trainings. Some members of staff, as the following quotes illustrates, did not get a chance to attend workshops, and hence felt that they were being unfairly treated, and thus felt demotivated.

*“The malaria case management workshop is a workshop that should also include EHTs as well and the coordination is also supposed to be done at the DHE level, but we are not invited. We need to get new knowledge and be innovative, things are changing, and how do you expect us to know new knowledge when we are not attending workshops”* [EHT].

While facility staff also reported other problems with their managers eg, allocation of tasks, sanctioning of leave days etc, the example of inability of some managers to manage training opportunities fairly, signals a lack of formal accountability structures and appropriate checks and balances and insufficient preparation of those assigned to be facility managers, to take on these management responsibilities. Many young RGNs and DMOs pointed out that they were only trained clinicians, and that they had had little training or guidance on management, let alone on the complexities of management of human resource and financial processes.

### Local Context: Local Politics, Community Characteristics and Geography

#### The Influence of Local Politics

The RBF arrangements seek to revive the HCCs with a view to encourage community ownership of services and community mobilisation for improving utilisation. This implies that the functioning of the HCC had an influence on the performance of the facilities, and at the same time required facility managers to communicate and establish good relationships with the HCC. In contrast, in the traditional input–based financing arrangements, this was neither required, nor relevant.

Previous evaluations have shown that most of the high performing RBF facilities had well-functioning HCCs that met regularly.^10^ They jointly identified issues and prioritised activities for improvement of facilities and services and worked closely with facility staff to address these issues. The well-functioning HCCs also took interest in the performance data of their facility, often comparing it with that of other clinics, and learning from the better performing facilities.

However, wherever there were poorly functioning HCCs, irrespective of the reasons, it undermined the ability of the facility management to take timely decisions. This indecisiveness undetermined the achievement of agreed results, and translated into loss of incentive related earnings at facility level and at the individual level; this frustrated the staff and undermined their motivation to perform. The reasons for poor functioning of HCCs were almost always rooted in the local social and political context. The latter, the local politics was cited by health facility staff and managers as being a big problem. Problems included: (1) Elite capture of the HCC; (2) Domination of HCCs by members who were not constructive and not interested in working towards a common goal, but more interested in exercising power over the facility staff; (3) Local community members and HCC members, being envious and resentful of the monies/incentives being earned by facility staff; (4) Political/ideological differences between some of the health facility staff and HCC members. Poorly functioning HCCs could paralyse facility operations in many ways; for example, rules require a quorum to approve expenses at the health centre – as the following quote from an NIC illustrates, a matter as simple as low attendance of HCC meetings meant that funds for routine expenses would not be released.

*“The HCC has 13 members but as you can see from the minutes of the past 6 months the members present are always less than 5. This is the problem”* [NIC].

#### Differences in Community Contexts Impacting RBF Achievements

Local level burden of disease patterns and health seeking patterns did not often sufficiently align with what was included in the RBF targets. This related to: (1) The type of diseases that were included in the targets; (2) The environmental conditions which often determined the major burden of disease in certain areas (eg, occurrence of snakebites and rabies in one area); (3) The expected occurrence due to seasonality of diseases (eg, malaria and diarrhoea); (4) The local customs around care seeking (eg, the custom that the first delivery should be at the wife’s family home, leading to facilities not meeting the target of male involvement in antenatal care). As the quote below illustrates, local disease patterns, related to the malaria season, and to periods with diarrhoeal diseases outbreaks and high incidence of acute respiratory infections, played a significant role in influencing utilization and quality of care.

*“Starting from December to May that’s the rainy season and we are very busy because that is the malaria season and we are treating a lot of malaria patients. In September and October there is an increase in diarrhoeal diseases and during May and June there are a lot of ARIs. Despite putting effort to clear the long queues sometimes we find it difficult to manage the work as we are few”* [NIC].

The RBF approach in Zimbabwe had yet to factor in these local contextual factors in its incentive awarding arrangements; not doing so meant that some facilities (and the staff there) received (unfairly) lower incentive payments all the time, or during certain times, compared to some other facilities. These local and community level contextual influences, given that they were beyond the influence of the facility manager, constrained the ability of many managers to effectively lead their team and to keep them motivated to perform well.

#### Geographical Context Hampering Achievements of Generic RBF Targets

In the same vein, generic RBF targets are/were set with the presumption that 60% of the population would utilize the health facility. However, facility location both positively and negatively affected the ability to reach set targets, for example through: (1) Being in a remote and sparsely populated area with target populations that faced access and transport challenges; (2) Being in proximity to referral facilities or to alternative facilities that offered more services or had more staff; or, (3) Being in proximity to a non-RBF district (increasing the number of clients in an RBF facility). When contextual factors beyond the clinic manager’s control, decreased the facilities earnings, the failure to reach targets led to frustration amongst staff; it also undermined the credibility of the manager to lead, to foster teamwork and to maintain staff motivation.

## Discussion

Both, proponents and critics, have called for a more critical examination of the RBF approach.^2,16-19^ Fritsche et al,^20^ consider the process of building the research evidence on results and performance-based financing arrangements, to be ‘work in progress’; they have called upon researchers to use a variety of methodologies to examine RBF implementation. Our study contributes to this scientific exercise at 2 levels: through exploring an operational dilemma in the specific context of RBF implementation in Zimbabwe, and through using the insights gained from the study to shed light upon how operational issues and management arrangements on the ground, shape health policy implementation.

### The Dilemma of Fall in Motivation

At the heart of the RBF arrangements is the assumption that it brings about conditions which motivate health workers to perform and to deliver results.^10,20^ Fritsche et al^20^ in their performance-based financing toolkit, explain that the regular ‘salary system provides fairly little additional financial motivation to provide services compared to other remuneration schemes’ (p 136), and that RBF introduces strong financial motivations, while still ‘relying on health workers’ internal motivation’ (p 53). The fall in motivation, in spite of substantial financial incentives (at individual and facility levels), and in spite of having greater autonomy to allocate facility level resources, was counterintuitive and problematic from a policy implementation perspective. This fall was all the more significant given that at baseline, motivation across all 8 constructs was higher in the RBF areas. Recent work by Lohmann et al^21^ suggests that RBF arrangements can positively influence health worker’s motivation in a variety of ways, but that implementation-related challenges and contextual factors, often existing health system issues, constrain the achievement of full motivating potential. Consistent with Lohmann et al,^21^ facility staff in our study also reported that the resources, autonomy and incentives from RBF spurred them to work harder to achieve targets. It was clear that contextual factors, primarily pre-existing health system issues, negated the motivational effects of RBF. Further, across all 8 constructs, at endline, motivation in the RBF areas had fallen lower than in the control areas, with the most significant falls in the ‘leadership’ and ‘teamwork’ related constructs. Our study showed that the capability of managers to exercise leadership and foster teamwork was hampered by the health system context, the influence of local politics on the health system, by individual management capacities as well as by the inability of the RBF design to align with the local geographical setting. This suggests that pre-existing management and institutional issues do not merely negate the motivating influence of RBF, unless systematically addressed, they have the potential to undermine the motivation of some frontline staff.

Our findings signal that if the potential of achieving newly introduced rewards is frustrated by structural constraints, the loss of the reward that could have been had, amplifies the sense of powerlessness and frustration amongst agents, and undermines their intrinsic motivation. If the loss of the possible reward occurs despite the agents working hard, and if the agents can see that the loss was due to structural constraints and management capabilities, both beyond their sphere of influence, motivation can fall to levels even lower than before the rewards were introduced.

### The Centrality of Contextualising Health Policy Interventions

Recent reviews of literature on performance-based financing arrangements in low and middle-income countries have emphatically highlighted the importance of context to the implementation of RBF programs.^2,18,22^ Renmans et al^18^ have also argued for the need for operational research to identify these contextual influences, with a view to tailor RBF programs to different contexts. Our findings re-affirm the importance of contextualising the introduction and implementation of RBF and other such health policy interventions and provide insights for contextualisation of the RBF program in Zimbabwe. Our findings reiterate many of the points made by earlier empirical studies from other parts of Sub-Saharan Africa. For example, Ssengooba et al,^16^ Paul et al,^23^ and Sieleunou et al^5^ have emphasised that the quality, structure and capacities of health systems where RBF programs are introduced, the local macro and micro politics, and, different and changing health priorities, affect the implementation of RBF programmes. To us, these commonalities signal an overarching theme which transcends contexts – that the success of health policy reforms is contingent upon the capacity of a health system to absorb these reforms.

Acknowledging these commonalities, in the following sub-sections we discuss our findings to extend the knowledge base on 2 linked fronts. We discuss how operational issues and management arrangements on the ground, shape health policy implementation. We make a case for health policy reforms to be grounded in and informed by a thorough analysis of whether the proposed changes can be absorbed by the health system; we discuss the importance of a deliberate and comprehensive institutional change management process to precede and accompany any health reform process. This discussion in many ways echoes and extends what Bhatnagar and George^24^ found in the context of introduction of RBF in Nigeria; that the scope of RBF alone “may not be sufficient to solve outstanding structural constraints, and (it) needs to be aligned with other health systems reforms to improve health worker motivation and performance.”

### Reflections on the Process of Reallocation of Decision Space

At the heart of the RBF enterprise is the notion of devolution of authority to primary and secondary level facilities. Under RBF, facilities have the autonomy to use the funds they generate to tackle the priorities they identify. The premise of the RBF arrangement is that a combination of monetary rewards, at individual level and collectively at facility level, based on results, together with the autonomy to use these funds for improving service delivery, leads to health workers being motivated to perform and to deliver. Our findings show that the ability of facility managers and staff to fulfil the above is constrained by how the process of reallocation of decision space, as Bossert^25^ calls it, is being operationalised. Based on our findings, these constraints operate at 2 levels: On *one level*, health facility managers and facility staff have been socialised in a system (training and professional system) which was input based and hierarchical, and with very little decision space at the facility level; they also continue to be situated in a system that for many parts remains as such. This socialisation and institutional context places limits on the ability of many facility managers and staff to imagine themselves exercising discretion and availing themselves to the newfound decision space. At *another level*, higher level managers have for decades also been socialised in a system (training and professional system) which was hierarchical, and where very little decision space was accorded to lower levels – our findings show that higher level managers also, and often with valid accountability related reasons, struggle to let go and cede decision space to lower levels. Our findings mirror Spisak and colleagues’^4^ conclusion that RBF design should be an iterative process with careful and deliberate consultation with the actors involved in the management and implementation of RBF. We extend their insight by highlighting that the process of reallocation of decision space across actors is a delicate enterprise. We contend that it requires patient and diligent action to flexibly and if necessary, iteratively transfer authority, and it simultaneously requires building capacities of those to whom authority is being transferred, to be thoroughly prepared to absorb the additional decision space.^26^ Our study signals that more effort is required on this front to ensure smooth roll out of the RBF arrangements in Zimbabwe, and that it needs to be explicitly addressed wherever RBF arrangements are being similarly implemented.

### Managing Change: Need to Identify Capacity Gaps and to Address These

RBF introduces a wide range of new institutional arrangements, new roles, new tasks, and new ways of doing things, for facility managers and district and provincial health management teams.^27^ Our findings expose how insufficient preparedness of people and processes for this change, undermines their ability to perform. Facility managers in the RBF scheme of things, unlike in input-based financing arrangements of the yore, are required to exercise much more discretion and leadership to achieve results, which, have tangible consequences for themselves and for their facility staff. That facility managers (in our study) had a dual role: as service providers and facility managers, further complicated matters. Without coaching and mentoring on how to play this dual role, these facility managers are unlikely to be able to navigate this dual role successfully, and they will continue to struggle to implement the RBF program, particularly the aspects that require innovation, exercise of autonomy and team-based decision making. It follows that higher-level managers - DMOs and provincial directors, need to have the capacity to coach and mentor facility manager – something that perhaps has not received the attention it deserves in Zimbabwe, yet. Qualitative studies done in Nigeria,^24^ Mozambique,^4,6^ and Cameroon^5^ affirm these reflections.

Our findings add that for RBF arrangements to work, these gaps need to be explicitly addressed, both at pre-service and in-service level. Based on our findings, we contend that the struggles of managers at the facility, district and provincial levels can be attributed to the absence of required leadership and management competencies: Cognitive intelligence, social intelligence and emotional intelligence,^28^ on one hand, and financial management competencies on the other. Asiri et al,^13^ Okello and Gilson,^29^ and Fritzen et al,^30^ have highlighted the importance of the role of managers in shaping the motivation and performance of the staff they manage. Fritzen et al^30^ add that managers can act to fulfil their management function provided they have the leadership and management competencies, decision-making space and tools and support from higher ups, to do so. Our study findings are consistent with this body of literature; study participants, both facility managers and staff, recognise gaps in their capacities to absorb the new roles and responsibilities, and its consequences. This is a systemic problem and strengthening health system wide human resource management capacities is thus clearly an area which deserves greater attention – it has the potential to substantially improve the RBF program performance in Zimbabwe; this insight is also relevant to other contexts which are considering implementing RBF arrangements.

### Managing Change: Putting, Explicit Change Management Processes in Place, Is Critical

Evidence also shows that mere capacity development at different levels is not enough; for such system wide reform, as RBF arguably is, to be effected, explicit organisational change management processes need to be put in place, across the system. Carefully designed processes, which take into account the interest and willingness of various actors to change,^28^ and which are aware of and constructively engage with potential bottlenecks and points of resistance, are required to accompany the changes in institutional arrangements. Having such change management processes in place will support managers and health workers in better understanding their new roles and responsibilities and will allow policy-makers and managers to better deal with resistance to change. While it is beyond the scope of this paper to dwell into this, several models are available to guide change processes; they have been extensively documented in the context of the broader literature on organisational change.^31-33^

### Issues Deserving Further Work: An Agenda for Research

Recent reviews and qualitative studies on the implementation of performance-based financing arrangements, have identified a range of questions that deserve the attention of researchers. Renmans et al,^18^ have made the case for researchers to examine RBF interventions as complex interventions being introduced in complex and open health system. Witter et al^34^ argue that policy-makers and researchers need to critically examine “dynamic linkages between context, process of development, design, implementation and effects and between different health system pillars.” Grittner^8^ and Gautier et al’s^1^ reviews, and Sieleunou et al’s^5^ policy analysis illustrate the importance of a careful assessment of the historical and relational contexts; their work shows the importance of gaining insight into the power dynamics, and the related forms of influence (financial, relational, ideational, network and knowledge based) when examining policy reforms. This study extends this research agenda on RBF arrangements, and on health policy implementation broadly, by highlighting some important research questions about management arrangements around policy implementation.

For example, health facility managers clearly struggled with their dual roles – of being a provider, and member of a small team working towards common rewards on one hand, and on the other, being the arbiter, whose decisions have clear and often immediate monetary consequences for other team members. While there is rich literature in management studies on the travails of the middle manager, little research has been done from which planners and policy-makers could draw insight to construct human resource management arrangements to allow managers in such situations to optimally play this dual role. There is also little research on the ways and means for programs and for lower level managers to approach and navigate community level, local politics – while striking a balance between competing accountability expectations. While there is some literature, particularly in relation to health system decentralisation, on considerations and strategies for transferring decision spaces to lower levels, there is little work on doing so in the context of RBF where the prospects of monetary gains/losses are an important additional factor to reckon with, both in terms of individual incentives as well as collective financial resources to improve service performance and to reach public health targets. It is likely that managers in the context of faith-based facilities (a substantial number in Zimbabwe, and Africa at large) and secondary and tertiary level facilities will have different experiences – as the organisational cultures in these settings are likely to be different; there is little insight yet on this subject. Gaining answers to these questions can, not merely help improve the RBF program implementation in Zimbabwe, the insight can also inform improvements in implementation in other settings.

## Limitations: On the Construct Well Being

Findings from the impact evaluation study by Nguyen et al^7^ which showed that health workers under the RBF arrangements showed statistically significant reduction across 3 aspects ‘Leadership,’ ‘Teamwork,’ and ‘Well Being’ were the trigger for this study. In the process of designing this study, and while testing the interview topic guides, we struggled with the notion of appropriately including lines of inquiry about ‘Well Being.’ We were faced with 2 major issues. One, the lines of inquiry which constituted the construct of well-being in Nguyen et al’s survey^7^ were deemed by study participants to be social experiences that were shaped less by the work environment, let alone one program, but largely by one’s private and social situation; participants further felt that these aspects were beyond the sphere of influence of the facility managers. Secondly, and in line with the views of the participants, and in discussions within the research team, it was realised that ‘Well Being’ was not at the same level as the other 7 constructs, and that well-being was perhaps better understood as the consequence of many, if not all the other constructs included in the survey. It is possible that the notion of well-being, as defined in the literature, is not a sufficiently valid construct when researching health worker motivation in the context of RBF in the Zimbabwean health system; it is however also possible, that we fell short with our lines of inquiry in eliciting it as such.

## Conclusion

This study clearly shows the importance of recognising RBF as a policy intervention being introduced in a complex environment with pre-existing dynamics. It is critical that those contemplating the introduction or scale up of RBF arrangements, or for that matter any complex health system reform, recognise that the process entails inserting complex relational dynamics within the larger health system; many of which are potentially disruptive of the status quo. This study illustrates the importance of thoroughly studying these relational dynamics, before, during a trial implementation phase, and on an ongoing basis. We argue that such a critical and reflective approach will allow funders and countries: to make a measured and realistic judgment about the feasibility of introduction and/or further scale up of RBF arrangements, and, where applicable, concomitant development of management processes to effectively absorb changes.

By carefully examining the effect of RBF arrangements on health worker motivation, this study shows that the implicit assumption of RBF-interventions that new institutional, financial and governance arrangements will ‘automatically’ improve leadership and teamwork to achieve motivation to perform better, cannot hold. It demonstrates the importance of analysing existing arrangements and capabilities and taking these into account when designing and implementing RBF interventions. We argue that RBF arrangements, by giving incentives, providing autonomy, and adapting management arrangements, cannot overcome chronic systemic weaknesses, on their own. A fall in motivation is but a prelude to a fall in performance; if system level problems and deficits are not addressed, the gains made thus far might not be maintained.

## Acknowledgements

The study was funded by financial support from the World Bank, and the implementation of the study was extensively supported facilitated by the Ministry of Health of Zimbabwe; contributions are duly acknowledged. However, the views expressed are solely of the authors.

## Ethical issues

Ethical approval for this study was obtained from Medical Research Council of Zimbabwe, Harare, Zimbabwe.

## Competing interests

All author’s time, except PM, was funded through a World Bank contract. Author PM’s time was funded by the Ministry of Health. The study was part of a process evaluation of a project being implemented in Ministry of Health facilities, with funding support from the World Bank.

## Authors’ contributions

SK, MD, RM, and PM conceptualised the study. SK, MD, CG, IM, and CS developed the study protocol and study tools. CG and IM collected the data. PM and RM supported data collection. SK, MD, CG, IM, and CS analysed the data. SK and MD with inputs from all co-authors, drafted and finalised this manuscript. All authors have reviewed the manuscript.

## Authors’ affiliations

^1^KIT Royal Tropical Institute, Amsterdam, The Netherlands. ^2^Nossal Institute for Global Health, Melbourne School of Population and Global Health, University of Melbourne, Melbourne, VIC, Australia. ^3^Independent Consultant. ^4^World Bank Group, Washington, DC, USA. ^5^Ministry of Health and Child Care, Government of Zimbabwe, Harare, Zimbabwe.

## Key messages

**Implications for policy makers**

- Introduction of Results-Based Financing (RBF) arrangements should be recognised as a system wide reform and change process.
- Policy-makers in low- and middle-income country contexts should allow the introduction of complex wide reforms only when they are assured of the capacity of their health systems to effectively absorb the many complex changes that are usually entailed in such processes.
- Leadership and teamwork are crucial determinants for health worker motivation, including within RBF arrangements. Successful RBF implementation requires that conditions are met to improve leadership and management at local facility level. For example, through: decentralisation of decision-making; clarity about roles and responsibilities; better support to local level managers in staff and stakeholder management.
- If RBF arrangements are being introduced, it is critical to recognise that they insert complex relational dynamics within the health system. These should be thoroughly studied during a trial implementation phase, and on an ongoing basis. Doing so will allow: adapting the RBF intervention to the context and a measured judgment about the feasibility of further scale up.
- For effective implementation of RBF arrangements, explicit organisational change management processes need to be put in place, across the health system.

**Implications for public**

Paying health workers based on performance is easier said than done. Zimbabwe’s experience shows that pay for performance schemes in the public health services can be effective, but also confirms the importance of taking into account the current system and capacities before initiating any changes. The case of Zimbabwe highlights that introduction of any such scheme requires careful reflection and diligent preparation, especially to enable local level managers to effectively play their role as leaders and supporting staff to work in teams. Findings show that not doing so is likely to negatively impact on health worker motivation.

## References

[1] Gautier L, Tosun J, De Allegri M, Ridde V (2018). How do diffusion entrepreneurs spread policies? Insights from performance-based financing in Sub-Saharan Africa. World Dev.

[2] Paul E, Albert L, Bisala BNS (2018). Performance-based financing in low-income and middle-income countries: isn’t it time for a rethink?. BMJ Glob Health.

[3] Bonfrer I, Van de Poel E, Van Doorslaer E (2014). The effects of performance incentives on the utilization and quality of maternal and child care in Burundi. Soc Sci Med.

[4] Spisak C, Morgan L, Eichler R, Rosen J, Serumaga B, Wang A (2016). Results-Based Financing in Mozambique’s Central Medical Store: A Review After 1 Year. Glob Health Sci Pract.

[5] Sieleunou I, Turcotte-Tremblay AM, Fotso JT (2017). Setting performance-based financing in the health sector agenda: a case study in Cameroon. Global Health.

[6] Schuster RC, de Sousa O, Reme AK (2018). Performance-Based Financing Empowers Health Workers Delivering Prevention of Vertical Transmission of HIV Services and Decreases Desire to Leave in Mozambique. Int J Health Policy Manag.

[7] Nguyen HTH, Gopalan S, Mutasa R, et al. Impact of Results-Based Financing on Health Worker Satisfaction and Motivation in Zimbabwe: Implications for program design and implementation. Washington, DC: World Bank; 2015.

[8] Grittner AM. Results-based Financing: Evidence from performance-based financing in the health sector. Bonn: German Development Institute; 2013.

[9] Musgrove P. Financial and Other Rewards for Good Performance or Results: A Guided Tour of Concepts and Terms and a Short Glossary. Washington, DC: World Bank; 2011.

[10] World Bank. Learning from Implementation: Process Monitoring and Evaluation II of Zimbabwe’s Results-Based Financing Project. The case of Mutoko, Chiredzi, Nkayi and Kariba districts. Washington: The World Bank Group; 2015.

[11] World Bank. Rewarding Provider Performance to Improve Quality and Coverage of Maternal and Child Health Outcomes. Zimbabwe Results-Based Financing Pilot Program: Evidence to Inform Policy and Management Decisions. Washington: The World Bank Group; 2016.

[12] Shen GC, Nguyen HT, Das A (2017). Incentives to change: effects of performance-based financing on health workers in Zambia. Hum Resour Health.

[13] Asiri SA, Rohrer WW, Al-Surimi K, Da’ar OO, Ahmed A (2016). The association of leadership styles and empowerment with nurses’ organizational commitment in an acute health care setting: a cross-sectional study. BMC Nurs.

[14] World Bank. Using results-based financing to achieve maternal & child health: Progress report. Health Results Innovations Trust Fund; 2013.

[15] Ritchie J, Spencer L. Qualitative data analysis for applied policy research. In: Bryman A, Burgess R, eds. Analyzing Qualitative Data. London: Routledge; 1994:173-194.

[16] Ssengooba F, McPake B, Palmer N (2012). Why performance-based contracting failed in Uganda--an “open-box” evaluation of a complex health system intervention. Soc Sci Med.

[17] Turcotte-Tremblay AM, Spagnolo J, De Allegri M, Ridde V (2016). Does performance-based financing increase value for money in low- and middle- income countries? A systematic review. Health Econ Rev.

[18] Renmans D, Holvoet N, Orach CG, Criel B (2016). Opening the ‘black box’ of performance-based financing in low- and lower middle-income countries: a review of the literature. Health Policy Plan.

[19] Mayaka Ma-Nitu S, Tembey L, Bigirimana E (2018). Towards constructive rethinking of PBF: perspectives of implementers in sub-Saharan Africa. BMJ Global Health.

[20] Fritsche GB, Soeters R, Meessen B. Performance-Based Financing Toolkit. Washington, DC: World Bank; 2014.

[21] Lohmann J, Wilhelm D, Kambala C, Brenner S, Muula AS, De Allegri M (2018). ‘The money can be a motivator, to me a little, but mostly PBF just helps me to do better in my job’ An exploration of the motivational mechanisms of performance-based financing for health workers in Malawi. Health Policy Plan.

[22] Meessen B, Shroff ZC, Bigdeli M (2017). From Scheme to System (Part 1): Notes on Conceptual and Methodological Innovations in the Multicountry Research Program on Scaling Up Results-Based Financing in Health Systems. Health Syst Reform.

[23] Paul E, Sossouhounto N, Eclou DS (2014). Local stakeholders’ perceptions about the introduction of performance-based financing in Benin: a case study in two health districts. Int J Health Policy Manag.

[24] Bhatnagar A, George AS (2016). Motivating health workers up to a limit: partial effects of performance-based financing on working environments in Nigeria. Health Policy Plan.

[25] Bossert T (1998). Analyzing the decentralization of health systems in developing countries: decision space, innovation and performance. Soc Sci Med.

[26] Bossert TJ (2016). Decision Space and Capacities in the Decentralization of Health Services in FijiComment on “Decentralisation of Health Services in Fiji: A Decision Space Analysis. ” Int J Health Policy Manag.

[27] Soeters R, Habineza C, Peerenboom PB (2006). Performance-based financing and changing the district health system: experience from Rwanda. Bull World Health Organ.

[28] Daire J, Gilson L, Cleary S. Developing leadership and management competencies in low and middle-income country health systems: a review of the literature. South Africa: Health Economics Unit, University of Cape Town; 2014.

[29] Okello DR, Gilson L (2015). Exploring the influence of trust relationships on motivation in the health sector: a systematic review. Hum Resour Health.

[30] Fritzen SA (2007). Strategic management of the health workforce in developing countries: what have we learned?. Hum Resour Health.

[31] Hendry C (1996). Understanding and creating whole organizational change through learning theory. Hum Relat.

[32] Beer M, Eisenstat RA, Spector B (1990). Why change programs don’t produce change. Harv Bus Rev.

[33] Muncer J, Kabwe C (2015). An evaluation of how change is managed in practice and the key considerations for organisations undertaking a change. Journal of Research Studies in Business & Management.

[34] Witter S, Toonen J, Meessen B, Kagubare J, Fritsche G, Vaughan K (2013). Performance-based financing as a health system reform: mapping the key dimensions for monitoring and evaluation. BMC Health Serv Res.

